# Early detection of general movements trajectories in very low birth weight infants

**DOI:** 10.1038/s41598-020-70003-3

**Published:** 2020-08-06

**Authors:** Matteo Porro, Camilla Fontana, Maria Lorella Giannì, Nicola Pesenti, Tiziana Boggini, Agnese De Carli, Giovanna De Bon, Giovanna Lucco, Fabio Mosca, Monica Fumagalli, Odoardo Picciolini

**Affiliations:** 1 Fondazione IRCCS Ca’ Granda Ospedale Maggiore Policlinico, Pediatric Physical Medicine & Rehabilitation Unit, Milan, Italy; 2 University of Milan, Department of Clinical Sciences and Community Health, Milan, Italy; 3 Fondazione IRCCS Ca’ Granda Ospedale Maggiore Policlinico, NICU, Milan, Italy; 4 Department of Statistics and Quantitative Methods, Division of Biostatistics, Epidemiology and Public Health, University of Milano-Bicocca, Milan, Italy

**Keywords:** Paediatric research, Paediatric neurological disorders

## Abstract

The aim of the study was to investigate General Movements’(GMs) neonatal trajectories and their association with neurodevelopment at three months corrected age (CA) in preterm infants. We conducted an observational, longitudinal study in 216 very low birth weight infants. GMs were recorded at 31 ± 1, 35 ± 1, 40 ± 1 weeks of postmenstrual age and at three months of corrected age (CA). More than 90% of infants showing neonatal trajectories with persistent Normal (N-N) or initial Poor Repertoire to Normal (PR-N) movements presented fidgety pattern at three months CA. On the contrary, fidgety movements were not detected in any infant with a trajectory of persistent Cramped-Synchronized (CS-CS) or an initial Poor-Repertoire to Cramped-Synchronized (PR-CS) movements. Trajectories with initial Normal to Poor-Repertoire (N-PR) or persistent Poor-Repertoire (PR-PR) movements showed an increased risk of having a non-normal Fidgety pattern compared with the N-N group (OR = 8.43, 95% CI: 2.26–31.45 and OR = 15.02, 95% CI: 6.40–35.26, respectively). These results highlight the importance to evaluate neonatal GMs’ trajectory to predict infants’ neurodevelopment. N-N or PR-N trajectories suggest normal short-term neurodevelopment, especially a lower risk of Cerebral Palsy; whereas findings of N-PR and PR-PR trajectories indicate the need for closer follow up to avoid delay in programming intervention strategies.

## Introduction

During the last twenty years survival rate of preterm infants has risen in high-income countries thanks to continuous research in perinatal care and innovative technologies^1^. However, concerns about their long-term outcome are still high. As a matter of fact, 5–10% of preterm infants born before the 32nd weeks of gestation suffer from major neurological disorders including Cerebral Palsy (CP) and severe intellectual disability; moreover a higher percentage, around 25–50% of preterm infants, suffer from minor neurodevelopmental delay^2^. Minor sequelae could manifest even in the absence of overt brain lesions and could affect neurodevelopment leading to motor (i.e. clumsiness or developmental coordination disorders), cognitive (i.e. mild cognitive delay, learning disabilities) and behavioral (i.e. autism, hyperactivity) impairments^3,4^.

Given the complexity of these frameworks, the diagnosis is often postponed to a period in which elementary skills emerge and develop. Nevertheless, early detection of neurodevelopmental disorders is of extreme importance to direct early intervention strategies^5^.

Prechtl’s General Movements (GMs) assessment is considered one of the most accurate tools to evaluate the integrity of the central nervous system. It can be used from birth until 20 weeks' post-term, it is a non-invasive and relatively easy-to-use assessment^6,7^. The GMs’ predictive validity for later neurodevelopmental outcome is well documented, particularly when assessed at three months corrected age^8,9^. Indeed, at this age, the absence of the normal fidgety pattern has a strong predictive value for CP^10^ and favoring the possibility for an early diagnosis and intervention. GMs’ quality is affected by gestational age at birth and several perinatal variables^11,12^. Furthermore most studies evaluating GMs trajectories have included small cohort of preterm infants^13,14^. As a result, there is a limited understanding of how GMs’ trajectories may evolve over time before term age^9^. Gaining further insight into GMs’ time-based changes could contribute to the early identification of infants at greater risk for adverse outcome, who could benefit from an early neurodevelopmental intervention^15^.

In the current study, we aimed to investigate GMs’ neonatal trajectories in a cohort of very low birth weight infants up to term equivalent age (TEA) and their relationship with the three months of corrected age (CA) GMs.

## Methods

### Subjects

The study was approved by the Ethics Committee Milano Area B. Parental informed consent was obtained from both parents.

All preterm infants with a birth weight less than 1,500 g, admitted to NICU, Fondazione IRCCS Ca’ Granda, Ospedale Maggiore Policlinico from January 2014 to April 2017, were enrolled in the study.

Infants affected by genetic syndromes and/or major congenital malformations were excluded.

All the infants, after discharge, entered the standard follow-up program that include: paediatric and neurofunctional evaluation and parental educational support.

### Study design

We conducted an observational, longitudinal study. Infants were enrolled after the first week of life, with clinical stable conditions, defined as no need of invasive mechanical ventilation and/or cardiovascular support, no active sepsis and no surgical pathologies.

The evaluation of infants’ qualitative spontaneous movements was conducted following the Prechtl’s GMs approach^6^. Each video-recording lasted about 5 min and contained at least three general movement sequences. Infants GMs were registered only in condition of clinical stability when the infant was in an alert behavioural state, with no pacifier, supine either in the crib or partially nested (with the exception of the three months CA recording).

Serial GMs were recorded to define neonatal GMs trajectories at three different timepoints: 31 ± 1 weeks of postmenstrual age (PMA), 35 ± 1 weeks of PMA, 40 ± 1 weeks of PMA. In the present study the short-term neurodevelopmental outcome is defined by the presence of the Fidgety pattern evaluated at 3 months CA.

GMs were scored in accordance with the Prechtl's qualitative assessment of GMs^6^, and therefore from 31 ± 1 to 40 ± 1 weeks PMA GMs were classified as normal (N), Poor Repertoire (PR) or Cramped Synchronized (CS), whereas at 3 months CA GMs were scored as Fidgety (F +), Abnormal Fidgety (AF) or Absent Fidgety (F −).

Scoring of the videos was performed independently by three investigators who were unaware of the infant’s clinical history and each assessor was blind to the scores given by the other two. All assessors had GMs certification from the GMs Trust. The final score was identified when there was an accordance by at least two assessors.

Longitudinal neonatal GMs' trajectories were described for each infant based, at least, on two of the three evaluations from 31 ± 1 to 40 ± 1 week PMA. Consequently, GMs' trajectories were classified and ranked for analysis as:

- Normal-Normal (N-N): the normal pattern was observed at all the neonatal GMs assessment;
- Persistence of poor repertoire pattern during the entire neonatal period (PR-PR): the PR pattern was described at all the assessment;
- Persistence of Cramped Synchronized movements at all the evaluations (CS-CS): the CS pattern was observed at all the evaluations;
- Normal—Poor Repertoire (N-PR): observation of the normal writhing phase followed by a PR pattern;
- Poor Repertoire—Normal (PR-N): early PR pattern followed by normal writhing phase;
- Poor Repertoire—Cramped Syncronized (PR-CS): observation of a PR pattern that modifies into CS movements;
- Cramped Syncronized—Poor Repertoire (CS-PR): a CS pattern followed by PR movements.

The baseline characteristics were collected from hospital charts. Recorded data included: gender, birth weight and gestational age at birth, small for gestational age (SGA), according to Fenton's growth chart^16^, twin birth, mode of delivery, Apgar scores at 1 and 5 min, Clinical Risk Index for Babies (CRIB)^17^, duration of hospital stay and postmenstrual age at discharge.

The following neonatal morbidities were considered: Retinopathy of Prematurity (ROP) ≥ 3°^18^, severe Bronchopulmonary dysplasia (BPD)^19^, all stages of Necrotizing Enterocolitis (NEC)^20^, sepsis defined as increased plasma levels of c-reactive protein associated with a positive blood culture.

Brain lesions were defined according to the combination of findings on both cranial Ultrasound (cUS) and brain Magnetic Resonance Imaging (MRI) performed according to the local clinical imaging protocol that included sequential cUS scans, from birth up to TEA at the following time-points: day 1, 3, 5, 7, 10, 14, 21 and then every fifteen days until TEA. Conventional brain MRI was performed only once at TEA. Severe brain lesions were defined as: intraventricular hemorrhage grade III–IV^21^ and/or posthemorrhagic ventricular dilation (PHVD) and/or focal cerebellar hemorrhage and/or cystic periventricular leukomalacia (cPVL) and/or white matter punctate lesions more than 6 (PWML) and/or brain malformations.

### Statistical analysis

Descriptive statistics of the demographic aspects of the infants and for each GMs' trajectory was provided; continuous variables were reported as mean ± standard deviation or median (and range or interquartile range) and categorical variables as overall counts (and percentages). The baseline characteristics were compared between groups using one-way ANOVA or Kruskal–Wallis test for continuous variables and Fisher’s Exact test for categorical variables. Tukey HSD and Dunn's post-hoc tests were performed following ANOVA and Kruskal–Wallis test, while for nominal variables was used Fisher’s pairwise test with Holm corrections for multiple comparisons.

The relationship between the GMs’ trajectories and the 3 months CA evaluation was studied using logistic regression analysis, focusing on differences between normal (namely the presence of Fidgety movements) versus non-normal (namely the presence of Abnormal Fidgety or the absence of Fidgety pattern). Gestational age and weight at birth, number of severe brain lesions and number of other severe comorbidities (including all stages of NEC, sepsis, ROP grade 3–4 and severe BPD) were taken into account as potential confounders, and crude and adjusted odds ratios (OR) were estimated along with 95% confidence interval (CI). All tests were two-tailed, values of p < 0.05 were considered significant. Statistical analyses were performed using R version 3.5.3 (R Foundation for Statistical Computing, Vienna, Austria).

### Interrater agreement

The interrater agreement between three assessors (CF, OP, MP) at each time-point of the study was assessed using Fleiss' Kappa statistic and ranged from 0.82 to 0.91.

## Results

A total of 258 infants were enrolled in the study between January 2014 and April 2017.

During NICU stay 10 infants died and 4 were transferred to another hospital before discharge. Moreover, 1 infant was excluded from the study as he was postnatally diagnosed having a genetic syndrome. Eleven infants were excluded from the analysis on GMs trajectories as only one evaluation was available before the three months assessment. Moreover, 16 infants were lost at the three months follow-up evaluation and therefore excluded from the study.

A total of 216 infants were included in the analyses as they had both neonatal GMs trajectories and the three months CA GMs evaluation.

The baseline characteristics of the cohort are summarized in Table 1.

**Table 1:** Baseline characteristics of the population included.

Demographic features	Overall (n = 216)
Gestational age at birth (weeks), mean ± SD	29.3 (2.3)
Birth weight (g), mean ± SD	1,117 (268)
Male, n (%)	97 (45)
Twins, n (%)	100 (47)
Monochorionic twins, n(%)	45 (45)
Apgar score at 1’, median (range)	7 (1–9)
Apgar score at 5’, median (range)	8 (3–10)
Cesarean section, n (%)	192 (90)
Small for gestational age, n (%)	42 (20)
Days of hospitalization, median (IQr)	61 (47; 87)
Severe brain lesion, n (%)	29 (13)
Other comobidities, n (%)	56 (26)
Maternal age, mean ± SD	34.6 ± 5.6

Severe brain lesions included: IVH grade III–IV and/or PHVD and/or focal cerebellar hemorrhage and/or cPVL and/or more than six PWML and/or brain malformations. Severe comorbidities included (all stages of NEC, severe ROP, severe BPD and sepsis).

At 31 ± 1 weeks, 72 GMs assessments were not performed as: 28 infants were born after 31 weeks of PMA, 26 were pharmacologically sedated and 18 were critically ill. The 144 GMs observed were scored as: 95 normal (66%), 49 Poor Repertoire (34%). None of the infant enrolled presented a Cramped-Synchronized pattern at this age.

At 35 ± 1 weeks, three GMs assessments were not evaluated due to technical-problem on the video recording. The 213 GMs observed were scored as: 142 normal (67%), 65 Poor Repertoire (30%) and six as Cramped-Synchronized (3%).

At 40 ± 1 weeks, 4 GMs assessments were not performed as three families missed the follow-up appointment and for one infant the assessment was not scorable due to technical-problem on the video recording. The 212 GMs observed were scored as: 142 normal (67%), 61 Poor Repertoire (29%) and 9 as Cramped-Synchronized (4%).

### General movements trajectories

We identified 6 different GMs trajectories between 31 ± 1 and 40 ± 1 weeks of PMA.

The baseline characteristics and short-term morbidities of infants across the different trajectories of GMs are reported in Table 2.

**Table 2:** Baseline characteristics and short-term morbidities across GMs trajectories.

Demographic features	N-N (n = 128)	PR-N (n = 17)	N-PR (n = 12)	PR-PR (n = 50)	PR-CS (n = 5)	CS-CS (n = 4)	p.value
Gestational age at birth (weeks), mean ± SD	30.0 (2.1)	28.4 (2.1)	29.6 (1.6)	28.1 (2.2)	27.5 (1.7)	27.6 (3.2)	< 0.001^a^
Birth weight (g), mean ± SD	1,188 (236)	1,006 (230)	1,117 (258)	996 (289)	954 (391)	1,030 (333)	< 0.001^a^
Male, n (%)	49 (38)	6 (35)	8 (67)	29 (58)	3 (60)	2 (50)	0.035^c^
Twins, n (%)	60 (47)	9 (53)	3 (25)	27 (55)	1 (20)	0 (0)	0.298^c^
Monochorionic twins, n(%)	28 (47)	6 (67)	2 (67)	8 (30)	1 (100)	0 (100)	0.164^c^
Apgar score at 1’, median (range)	7 (1–9)	7 (2–8)	8 (4–9)	6 (2–9)	5 (3–7)	4 (1–6)	< 0.001^b^
Apgar score at 5’, median (range)	8 (5–10)	8 (5–9)	9 (6–10)	8 (6–10)	7 (6–8)	8 (3–9)	0.032^b^
Cesarean section, n (%)	115 (91)	13 (76)	9 (75)	46 (94)	5 (100)	4 (100)	0.060^c^
Small for gestational age, n (%)	27 (21)	2 (12)	2 (17)	10 (20)	1 (20)	0 (0)	0.882^c^
Days of hospitalization, median (IQr)	55 (44; 70)	72 (56; 84)	68 (59; 84)	99 (54; 127)	92 (79; 99)	96 (78; 147)	< 0.001^b^
Severe brain lesion, n (%)	5 (4)	2 (12)	2 (17)	11 (22)	5 (100)	4 (100)	< 0.001^c^
Other comorbidities, n (%)	20 (16)	4 (24)	3 (25)	23 (46)	3 (60)	3 (75)	< 0.001^c^
Maternal age, mean ± SD	35.0 (5.3)	32.1 (6.7)	33.2 (7.5)	34.8 (6.7)	36.8 (4.1)	30.3 (5.7)	0.237^a^

Persistence of normal pattern (N-N), early poor repertoire followed by a normal pattern (PR-N), early normal followed by a poor repertoire pattern (N-PR), persistence of poor repertoire pattern (PR-PR), early poor repertoire followed by cramped synchronized pattern (PR-CS), persistence of cramped synchronized pattern (CS-CS).

^a^One way ANOVA.

^b^Kruskal Wallis test.

^c^Fisher’s exact test.

The most represented is the N-N trajectory (59.3%), namely a persistence of normal pattern. The trajectories that included CS movements were the less likely to occur (2.3% had a CS-CS trajectory while 1.9% had a PR-CS trajectory). The 23% of the entire population showed a PR-PR trajectory, therefore a persistence of poor repertoire movements during the entire neonatal period.

Taking into account only the infants that exhibit a trajectory either N-PR, PR-N or PR-PR, those with a persistence of the PR pattern (PR-PR) were the ones that presented the most severe neonatal morbidities (severe ROP, NEC, severe BPD and sepsis) and had an higher number of severe brain lesions. The infants that showed a normalization of the GMs pattern after the observation of a PR pattern (PR-N) had a shorter, even if not significant, median hospital stay compared to the PR-PR infants (72 versus 99 days) (Table 2).

Overall, the infants with a CS-CS trajectory were more likely to have suffered multiple neonatal morbidities and severe brain injury. Similarly, this occurred in the infants with a PR-CS trajectory (Table 2).

On the other hand, the infants in the N-N group, compared to those with PR-PR and PR-N trajectories, had the highest gestational age (p < 0.001 and p = 0.021, Tukey HSD) and weight at birth (p < 0.001 and p = 0.028, Tukey HSD). However, infants in the N-N group compared to PR-PR group had shorter median hospital stay (p < 0.001, Dunn’s test) and were less likely to develop severe brain lesions (p = 0.003, Fisher’s test) and other morbidities (p < 0.001, Fisher’s test) (Table 2).

At 3 months CA, the majority of infants showing a persistent normal GMs pattern (N-N) and a PR-N neonatal trajectory had fidgety movements (92% and 94% respectively); on the contrary, infants showing either CS-CS or a PR-CS neonatal trajectory did not have fidgety movements. Moreover, infants showing either a PR-PR or a N-PR trajectory showed fidgety movements only in half cases (50% and 52% respectively).

Detailed distribution frequency of the 3 months GMs outcome for each neonatal trajectory is reported in Table 3.

**Table 3:** Distribution frequency of the 3 months GMs outcome for each neonatal trajectory.

Trajectory	3 months follow up-assessment		
	Fidgety (F +) n (%)	Abnormal fidgety (AF) n (%)	Absent fidgety (F −) n (%)
N-N	117 (92)	10 (8)	0 (0)
PR-N	16 (94)	1 (6)	0 (0)
N-PR	11 (52)	6 (29)	4 (19)
PR-PR	24 (50)	17 (35)	7 (15)
PR-CS	0 (0)	2 (40)	3 (60)
CS-CS	0 (0)	0 (0)	4 (100)

Persistence of normal pattern (N-N), early poor repertoire followed by a normal pattern (PR-N), early normal followed by a poor repertoire pattern (N-PR), persistence of poor repertoire pattern (PR-PR), early poor repertoire followed by cramped synchronized pattern (PR-CS), persistence of cramped synchronized pattern (CS-CS).

Results of the logistic regression model between neonatal trajectories and risk of non-normal Fidgety pattern (namely Abnormal Fidgety and Absent Fidgety) at three months are reported in Table 4.

**Table 4:** Logistic regression model’s estimates.

Trajectory	OR	CI 95%	OR	CI 95%
	Crude		Adj	
PR-N	0.74	0.09–6.15	0.67	0.08–5.86
N-PR	8.43	2.26–31.45	8.05	2.12–30.62
PR-PR	15.02	6.40–35.26	11.10	4.34–28.34

Odds ratios (OR), estimated along with 95% confidence interval (CI), represents the risk of non-normal fidgety pattern (namely AF and F −) at 3 months. Persistence of normal pattern (N-N) trajectory used as reference. Adjusted for: gestational age at birth, birth weight, number of severe brain lesions and number of severe comorbidities. Trajectories definitions: early poor repertoire followed by a normal pattern (PR-N), early normal followed by a poor repertoire pattern (N-PR), persistence of poor repertoire pattern (PR-PR).

The N-PR and PR-PR trajectories showed an increased risk of having a non-normal Fidgety pattern compared with the N-N group (OR = 8.43, 95% CI: 2.26–31.45 and OR = 15.02, 95% CI: 6.40–35.26, respectively). No significant differences were observed between N-N and PR-N trajectories. Model’s estimates for the PR-CS and CS-CS’s trajectories are not feasible because all infants in these groups present not-normal fidgety at three months (78% of these infants present a F-pattern), therefore were excluded from the model.

## Discussion

The findings of this study allow to gain further insight into GMs’ trajectories in very low birth weight infants and suggests that the observation of GMs trajectories may be a useful tool in NICU to identify early neurodevelopmental outcomes.

In our population the N-N and PR-N trajectories appear to indicate normal neurodevelopment outcome at three months of CA whereas CS-CS or PR-CS trajectories suggest a higher risk for non-normal GMs assessment at three months of CA. In particular, the trajectories here proposed allow to have an early understanding of infant’s early outcome and this is of paramount importance as it is well known how the absence of fidgety is a strong predictor for CP^10^.

The early identification of infants at a greater risk to later neurodevelopmental delay would allow to target early intervention strategies to those most at risk. In fact early developmental intervention aims to improve brain connectivity during key periods of brain development, rather than waiting for an impairment to occur once altered brain connection have developed, highlighting the importance not to miss the first phase of infants neurodevelopment ^22–24^.

On the other hand infants showing a normal pattern throughout the neonatal period are more likely to present Fidgety movements at the 3 months CA assessment (92% with a neonatal N-N pattern): the identification of these infants would allow a better allocation of neurodevelopmental therapy that could be directed to those most at risk on the basis of a neurodevelopmental driven criterion in addition to the medical characteristics of the infant^25^.

With respect to the PR pattern our study confirms that the predictive validity of a single observation of poor repertoire movements is low when assessed only in the neonatal period and it is therefore crucial to assess GMs at different timepoints^26^.

In fact, for infants with a PR pattern, when looking at their neonatal trajectories, only those that present a normalization in the quality of GM by TEA, namely those with a PR-N trajectory, exhibit the Fidgety pattern at the 3 months assessment in the majority of the cases (94%).

Instead those infants that presented either a N-PR or a PR-PR trajectory have a higher risk to exhibit an abnormal GMs pattern at 3 months CA (48% and 50% respectively), with half of them displaying an abnormal fidgety or an absence of the fidgety.

These results can be partially explained when looking at the neonatal comorbidities, as infants showing a persistence of the PR pattern have higher incidence of sepsis, severe BDP, severe brain lesions and a longer hospital stay. Consistently, Olsen et al. that reported abnormal GMs association with the presence of severe neonatal comorbidities as IVH grade III-IV and sepsis^27^. In particular the clinical presentation of sepsis is often associated with hypokinesia and a poorer quality of GMs has been related to this condition in the acute phase^28^. However, even if in our study the infants were not evaluated during the acute phase, we observed a higher persistence of the PR pattern in these infants, as previously described by Beccaria et al.^29^.

Furthermore, in the present study, neonates developing trajectories including CS pattern had a high incidence of neonatal comorbidities and all of the infants presented brain lesion. Accordingly, previous studies in literature observed the influence of acute perinatal factors on brain function, with early changes of the central pattern generators of GMs^30^. The results of our study confirm how brain lesions are present in all the infants that exhibit a CS pattern at any stage^31^ as severe brain injury remains the primary responsible for adverse neurodevelopmental outcome in very preterm infants^32^.

In contrast to other studies, no transient CS trajectories were observed in our population^27^ as the CS pattern was either present at all the GMs assessment (CS-CS) or emerged after an observation of the PR pattern and remained stable until TEA (PR-CS).

In line with other studies, infants with N-N trajectories had a limited incidence of perinatal comorbidities^33^, that could allow the emergence of normal pattern.

The role of comorbidities in affecting neurodevelopmental outcome is widely acknowledged and the present study confirms the importance to evaluate neonatal factors together with GMs trajectories to better identify infants at risk for neurodevelopmental disorders. Our study highlights the need to take into account the role of comorbidities especially for those infants that display a PR pattern in the neonatal period. Furthermore, the independent association between persistence of PR pattern in GMs trajectories and later adverse outcome at three months of age supports the hypotheses of the importance of assessing GMs trajectories in preterm infants.

The main limitation of the study is that, although a relatively large cohort of very low birth weight infants enrolled, the number of pathological GMs patterns observed is limited, underlining the need for a multicentre study in order to confirm our results. Moreover, to better evaluate the outcomes of these infants, a longer follow-up and other neurodevelopmental tests are needed, as the predictive validity of the GMs assessment is wider in conjunction with other testing.

The current study confirms the need to consecutively evaluate GMs rather than having a single assessment to identify pattern of normal or atypical neurodevelopment in preterm infants. Moreover, the present study describes the individual GMs trajectories with several evaluation during the neonatal period and add knowledge to the predictive value of the neonatal GMs trajectory whereas the predictive value of GMs at three months of age is widely acknowledged^10^.

## Conclusion

The results of the present study highlight the importance of an accurate early GMs’ trajectory assessment, in order to evaluate infants’ neurodevelopment. Findings of N-N or PR-N trajectories may help clinicians in reassuring parents on normal short-term neurodevelopment of their infants, especially for a lower risk of CP. On the contrary, findings of CS-CS and PR-CS trajectories at term equivalent age indicate the need for clinicians to refer the infant to neurodevelopmental intervention during NICU stay whereas findings of N-PR and PR-PR trajectories indicate the need for closer follow up in order to avoid delay in programming potential intervention strategies.

The final perspective of this research is to guide clinicians in communication to parents the pathways and timing of early intervention during NICU stay and follow-up.
